# Safety and efficacy of anti-PD-1 in patients with baseline cardiac, renal, or hepatic dysfunction

**DOI:** 10.1186/s40425-016-0166-5

**Published:** 2016-10-18

**Authors:** Bridgette A. Kanz, Megan H. Pollack, Romany Johnpulle, Igor Puzanov, Leora Horn, Alicia Morgans, Jeffrey A. Sosman, Suthee Rapisuwon, R. Martin Conry, Zeynep Eroglu, Douglas B. Johnson

**Affiliations:** 1 Departments of Pharmaceutical Services, Vanderbilt Ingram Cancer Center, 2220 Pierce Avenue, Nashville, TN 37232 USA; 2 Departments of Medicine, Vanderbilt Ingram Cancer Center, 777 PRB, 2220 Pierce Ave., Nashville, TN 37232 USA; 3 Department of Medicine, Roswell Park Cancer Institute, Elm @ Carlton Streets, Buffalo, NY 14263 USA; 4 Department of Oncology, Georgetown Lombardi Cancer Center, 3970 Reservoir Road NW, Washington, DC 20007 USA; 5 Department of Medicine, University of Alabama at Birmingham, 1824 6th Avenue S, Birmingham, AL 35233 USA; 6 Department of Cutaneous Oncology, Moffitt Cancer Center, 12902 USF Magnolia Drive, Tampa, FL 33612 USA

**Keywords:** Anti-PD-1, Nivolumab, Pembrolizumab, Melanoma, Organ, Dysfunction, Cardiac, Renal, Hepatic

## Abstract

**Background:**

Anti-PD-1 therapy is increasingly used in various advanced malignancies. Patients with baseline organ dysfunction are largely excluded from clinical trials. Therefore it is unclear whether anti-PD-1 therapy is safe or effective in this setting. Further, these patients are often not candidates for other anti-cancer therapies, highlighting their need for active treatment options.

**Methods:**

We performed a retrospective analysis of patients from multiple centers with advanced solid tumors and baseline organ dysfunction who received anti-PD-1 therapy. Organ dysfunction was defined as cardiac (left ventricular ejection fraction ≤45 %), renal (creatinine ≥2 mg/dL or GFR ≤30 ml/min) or hepatic dysfunction (evidence of cirrhosis on imaging or AST, ALT or bilirubin ≥3x ULN). We assessed change in organ dysfunction, immune related adverse events (irAEs), response rate, progression free survival (PFS) and overall survival (OS).

**Results:**

We identified 27 patients eligible for inclusion with the following diseases: renal cell carcinoma (*n* = 8), melanoma (10), non-small cell lung cancer (3), small cell lung cancer (2) and urothelial bladder cancer (4). Baseline organ dysfunction included renal dysfunction (*n* = 17), hepatic dysfunction (7), cardiac dysfunction (11), including >1 organ dysfunction (8). Worsening organ dysfunction requiring hospitalization or dose delays occurred in 8 patients (30 %) although in most cases this was thought not-drug related and resolved with supportive care. Grade 3 irAEs occurred in 2 pts (7 %; hepatitis and colitis). Thirteen of 27 patients had ongoing treatment benefit (objective response or stable disease) at data collection (48 %). Eleven patients had primary progressive disease (41 %), 11 had stable disease (41 %), 4 had partial responses (15 %), and one had a complete response (4 %). Overall, median PFS was 168 days. Median OS was not reached.

**Conclusions:**

In our experience, anti-PD-1 agents in this group of patients with cardiac, hepatic or renal dysfunction were associated with tolerable irAEs and infrequent manageable worsening of organ dysfunction. Further, objective responses and prolonged PFS were observed in a number of patients. Thus, patients with baseline organ dysfunction may be considered for anti-PD-1 therapy with appropriate clinical monitoring.

## Background

Agents that block the interaction between programmed death-1 receptor and its ligand (PD-1/PD-L1) inhibit this negative immune regulator and thereby unleash anti-tumor immune responses. These agents, including nivolumab and pembrolizumab, have produced objective responses, many of which are durable, in numerous solid and hematologic malignancies [1–8]. As these agents are incorporated into standard treatment algorithms, questions have arisen regarding their use in patient populations excluded from clinical trials, including those with organ dysfunction.

Advanced malignancies have traditionally been treated with cytotoxic chemotherapy. Unfortunately, many patients have limited baseline organ function and/or performance status and are unable to receive optimal chemotherapy regimens. As such, they may have extremely limited therapeutic options. Due to their pharmacokinetic and pharmacodynamic properties, cytotoxic chemotherapies frequently result in bone marrow suppression, kidney and liver dysfunction, and cardiac toxicity. In patients that are further limited by baseline organ dysfunction, chemotherapy may exacerbate existing organ injury or result in decreased clearance or metabolism, increasing toxicities. By contrast, monoclonal antibodies are metabolized to peptides and amino acids by circulating phagocytic cells or by their target antigen-containing cells, rather than through the liver and kidneys [9]. Monoclonal antibodies bound to protective receptors are protected from degradation, explaining their exceptionally long half-lives [10].

These pharmacologic and immune properties result in a distinct toxicity profile specific to immunotherapies. These idiosyncratic adverse events, termed immune related adverse events (irAEs), include colitis, pneumonitis, hepatitis, nephritis, and endocrinopathies [11]. Although usually manageable with corticosteroids, irAEs represent a significant cause of morbidity and rarely mortality.

The effects of anti-PD-1 in patients with baseline organ dysfunction have not been explored. We hypothesized that, based on their pharmacokinetics and mechanism of action, anti-PD-1 therapies would not induce worsening organ dysfunction or high rates of irAEs in this population. To address, we performed a retrospective analysis of patients from four centers with pre-existing organ dysfunction treated with anti-PD-1 agents. We assessed safety endpoints, including irAEs and worsening organ dysfunction, and efficacy endpoints.

## Methods

### Patients

Eligible patients were 18 years of age or older, had a confirmed advanced malignancy, and had received at least one dose of either pembrolizumab or nivolumab between 6/1/2013 and 12/31/2015. Further inclusion criteria included access to medical records and baseline organ dysfunction. Organ dysfunction was defined as the following: 1) creatinine ≥2 mg/dL or estimated glomerular filtration rate (eGFR) ≤ 30 mL/min; 2) aspartate aminotransferase (AST), alanine aminotransferase (ALT), or total bilirubin ≥ 3 times institution upper limit of normal, or evidence of cirrhosis on imaging studies; and 3) ejection fraction ≤ 45 %. These particular organ systems were chosen since they often preclude clinical trial enrollment when dysfunctional. Dysfunction of other organs, including lungs, bone marrow, and brain were not assessed due to less objective measurements, less routine monitoring, and multifactorial nature of dysfunction. The cutoffs for particular organ dysfunction were somewhat arbitrary, but would often exclude patients from many clinical trials and/or from various chemotherapy regimens. Key exclusion criteria included patients who received combination immunotherapy with multiple agents (e.g. ipilimumab + nivolumab).

### Study design

This was a multicenter, retrospective analysis of patients with baseline organ dysfunction who received either pembrolizumab or nivolumab for the treatment of advanced malignancies. We collected patient demographics, organ function, medical co-morbidities, and Eastern Cooperative Group performance status (ECOG PS) at baseline.

### Outcomes

Safety endpoints were (1) new or progressive organ dysfunction defined as baseline dysfunction above, (2) hospitalization rate, and (3) adverse events as defined in the National Cancer Institute Common Terminology Criteria for Adverse Events (CTCAE version 4.03). Efficacy outcomes were (1) progression-free survival (PFS), (2) overall survival (OS), and (3) response rate per Response Evaluation Criteria in Solid Tumors (RECIST) version 1.1.

### Statistical analysis

Baseline demographics and treatment characteristics were analyzed using descriptive statistics, listed with frequencies and percentages for categorical variables and medians and ranges for continuous variables. PFS was defined as time from first dose of anti-PD-1 therapy to progression of disease via scans or documentation of progression in provider notes. OS was defined as the time from first dose of therapy to death from any cause. All surviving patients were censored at the time of last follow up. Survival distributions were estimated using the Kaplan-Meier method.

## Results

### Patients

A total of 27 patients with advanced malignancies and baseline organ dysfunction were included. Eight (30 %) had renal cell carcinoma (RCC), 10 (37 %) had melanoma, 3 (11 %) had non-small cell lung cancer (NSCLC), 2 (7 %) had small cell lung cancer (SCLC), and 4 (15 %) had urothelial bladder carcinoma. Most patients were male with a median age of 69, and a majority received nivolumab as their anti-PD-1 therapy. The median ECOG PS was 1 with a range from 0–3. Baseline patient characteristics are shown in Table 1. The majority of patients received ≥2 prior lines of treatment before anti-PD-1 therapy (*n* = 20, 74 %). The prevalence of baseline organ dysfunction was as follows: renal (*N =* 17, 63 %), hepatic (*N =* 7, 26 %), and cardiac (*N =* 11, 41 %) dysfunction. Eight (30 %) patients had dysfunction in multiple organ systems at baseline. All patients had comorbidities in addition to cancer.

**Table 1:** Baseline characteristics

Baseline features (*n* = 27)	N (%)
Median age, years (range)	69 (47–85)
Male	23 (85)
≥2 prior therapies	20 (74)
Organ dysfunction	
Renal dysfunction	17 (63)
Hepatic dysfunction	7 (26)
Cardiac dysfunction	11 (41)
2 organ dysfunctions	8 (30)
Received nivolumab	16 (59)
Median number of doses (range)	7 (1–52)
Disease state	
Renal cell carcinoma	8 (30 %)
Melanoma	10 (37 %)
Non-small cell lung cancer	3 (11 %)
Small cell lung cancer	2 (7 %)
Urothelial cell carcinoma	4 (15 %)

Among 17 patients with renal dysfunction, 3 were on hemodialysis or peritoneal dialysis. Causes of renal dysfunction were diverse, but included diabetes, hypertension, prior therapies (tyrosine kinase inhibitors), and nephrectomy (for RCC). Median creatinine was 2.7 (range 2–7.2 in the setting of hemodialysis). Among 7 patients with hepatic dysfunction, 4 had evidence of cirrhosis on imaging, including 2 with splenomegaly and gastroesophageal varices. Cirrhosis was caused by alcohol ingestion (*n* = 3) and hepatitis C (*n* = 1). Two patients had elevated bilirubin (baseline of 2.1 and 4.0 respectively), and two patients had elevated liver function tests (AST 123–150), all caused by metastatic disease. Among 11 patients with cardiac dysfunction, ejection fraction ranged from 10 to 45 %. Causes of cardiac dysfunction were primarily due to ischemia but also included alcoholic cardiomyopathy (*n* = 1) and several patients with hypertension and prior tyrosine kinase inhibitors in addition to coronary artery disease.

### Safety

Following anti-PD-1 therapy, irAEs were uncommon; two patients (7 %) experienced grade 3 irAEs, accounting for three total events (Table 2). One patient had metastatic RCC with baseline renal (creatinine 3.3) and cardiac dysfunction (ejection fraction 25–30 %) experienced grade 3 hepatitis that resolved with corticosteroids (prednisone 1 mg/kg tapered over 1 month). The other patient had NSCLC with a history of follicular lymphoma and renal dysfunction at baseline (baseline creatinine 4.6), and experienced grade 3 hepatitis and colitis which emerged concurrently and rapidly resolved with high-dose (1 mg/kg) prednisone. This patient discontinued therapy and was the only patient to do so due to toxicities.

**Table 2:** Immune-related adverse events

irAE	Grades 1/2 (N, %)	Grades 3/4 (N, %)
Arthralgias	1 (4 %)	0
Colitis	0	1 (4 %)
Conjunctivis	1 (4 %)	0
Diarrhea	1 (4 %)	0
Hepatitis	0	2 (7 %)
Hypothyroidism	4 (15 %)	0
Nephritis	2 (7 %)	0
Pruritis	1 (4 %)	0
Rash	3 (11 %)	0
Vitiligo	1 (4 %)	0

Next, we assessed the effects of anti-PD-1 treatment on patients’ baseline organ dysfunction. We did not observe irAEs leading to obvious worsening of patient’s organ function. Worsening organ function occurred in eight patients (30 %, Table 3), all thought to be unrelated to anti-PD-1 therapy. Three patients with baseline renal dysfunction experienced low-grade acute kidney injury, thought to be related to volume depletion (either due to decreased oral intake or excess diuresis). These resolved with hydration or adjustment of diuretic doses and did not require corticosteroids (e.g. unrelated to anti-PD-1 therapy by provider assessment). Five patients (4 with baseline congestive heart failure, 1 with cirrhosis) experienced volume overload, including four requiring hospitalization for diuresis. All of these cases occurred within the first 12 weeks of therapy. These did not recur with adjustment of oral diuretic doses, with or without concurrent use of intravenous diuretics during anti-PD-1 infusion. One case of refractory ascites of unclear etiology developed in a patient with metastatic RCC (but without significant abdominal involvement) and baseline kidney dysfunction (creatinine 2.2) requiring repeated paracentesis that was considered unrelated to anti-PD-1 therapy. An immune-related etiology was briefly considered, but no improvement was noted with prednisone. Notably, performance status appeared similar in patients who experienced organ dysfunction exacerbations and those who did not (ECOG 1 in the vast majority of cases).

**Table 3:** Organ dysfunction in individual patients

Baseline organ dysfunction	Baseline measurements	Worsening dysfunction (grade)
Renal and hepatic	Creatinine 2.6, cirrhosis on imaging	Edema (1)
Renal	Creatinine 3.0	Acute kidney injury (2)
Renal	Creatinine 2.2	Ascites (3)
Renal	Creatinine 4.6	Acute kidney injury (1)
Renal and cardiac	Creatinine 2.6, Ejection fraction 40 %	Acute kidney injury (1), volume overload (3)
Cardiac	Ejection fraction 30 %	Decompensated heart failure (3)
Cardiac	Ejection fraction 20 %	Decompensated heart failure (3)
Liver and cardiac	Ejection fraction 45 %, cirrhosis on imaging	Volume overload and decompensated heart failure (3)

To further assess the safety of anti-PD-1 therapy in this group of patients with significant medical co-morbidities, we evaluated whether patients were hospitalized following anti-PD-1. Ten (37 %) patients were hospitalized in the three months after the start of therapy, including 5 for organ dysfunction, and 5 for other reasons, primarily sepsis and syncope. Notably, at least 6 patients (22 %) were also hospitalized within 3 months prior to therapy (albeit with incomplete records in some cases); thus the attribution of anti-PD-1 for these findings is unclear.

### Efficacy

**Fig. 1:**
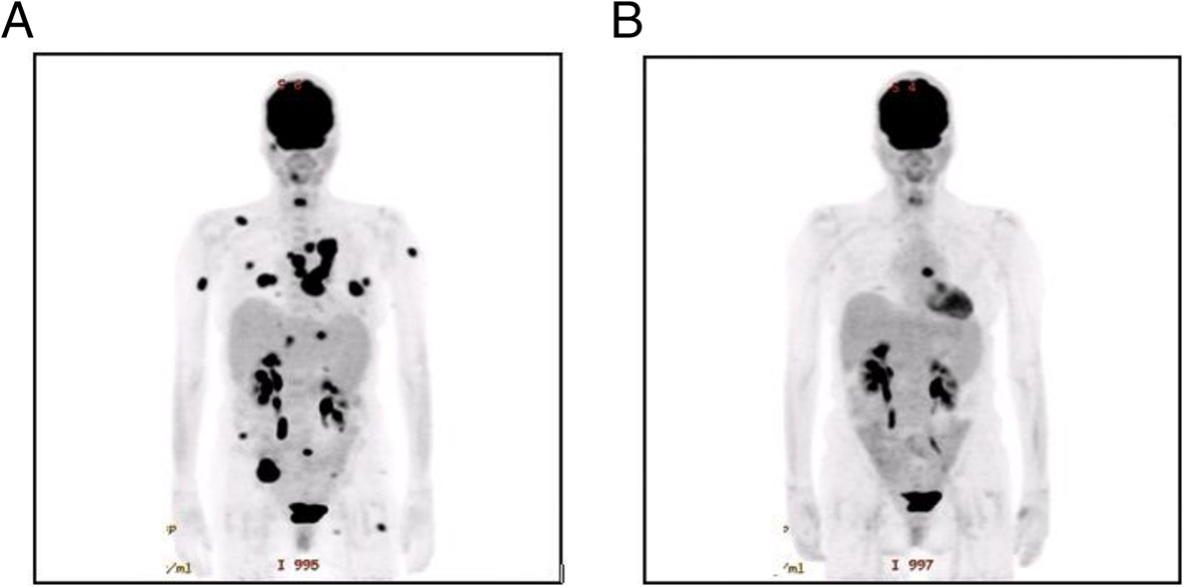
PET-CT for a patient with metastatic melanoma and baseline cirrhosis of the liver (**a**) and near complete response four months after initiating treatment (**b**)

**Fig. 2:**
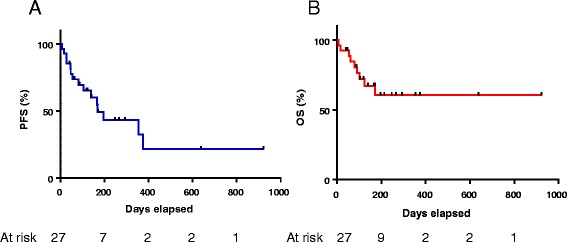
Progression free (**a**) and overall survival (**b**) among the study population (median duration of follow-up 139 days)

Thirteen patients (48 %) experienced ongoing treatment benefit (partial response or stable disease) at the time of data collection (Table 4). Eleven patients (41 %) had primary progressive disease, 11 (41 %) had stable disease as best response, 4 (15 %) had partial responses, and one (4 %) had a complete response. The patient with a complete response received pembrolizumab for metastatic melanoma and had baseline renal dysfunction at baseline. Other partial responders included two patients with melanoma (one with baseline renal dysfunction and one with cirrhosis; Fig. 1a, b), a patient with RCC, and a patient with NSCLC (both with baseline renal dysfunction). These patients had ECOG PS ranging from 0 to 3, and all had only a single organ dysfunction. Notably, the only patient categorized as having an ECOG PS 3 was the complete responder with melanoma. Among 3 patients on dialysis, none responded to therapy, although none of these patients experienced major toxicities either. Six patients (22 %) experienced rapid progression and death within 60 days of treatment initiation. Compared to the remainder of the cohort, these patients did not appear to have particularly poor performance status (all with ECOG PS listed as 1 by treating providers), or particularly severe organ dysfunction (2 of 6 with multiple organs involved, 1 of 6 on hemodialysis). The median PFS in this cohort was 168 days, and the median OS was not reached (Fig. 2).

**Table 4:** Response to therapy

Response	N (%)
Complete response	1 (4 %)
Partial response	4 (15 %)
Stable disease	11 (41 %)
Progressive disease	11 (41 %)

## Discussion

In this study, use of anti-PD-1 therapy in patients with baseline organ dysfunction resulted in rates of irAEs similar to previously reported clinical trials including patients without organ dysfunction. In this heavily pre-treated population with multiple comorbid illnesses, we demonstrate encouraging PFS and OS. Notably, exacerbation of baseline organ dysfunction was relatively uncommon, and was reversible with standard supportive care (e.g. diuresis or intravenous fluid administration). We speculate that the volume of fluid given with anti-PD-1 therapy may have contributed to progression of organ dysfunction, although we cannot rule out a drug-specific effect. While these data are immature, they suggest relative safety and efficacy in this patient population without excess risk of irAEs that resulted in worsening organ function. When taken in context with the favorable pharmacokinetics of pembrolizumab and nivolumab, anti-PD-1 therapies are a viable treatment option for this population.

Patients with baseline organ dysfunction represent a population that may be more susceptible to adverse events and is commonly excluded from clinical trials. These patients may be unable to tolerate traditional cytotoxic chemotherapy regimens due to organ dysfunction and poor performance status. Because of this, treatment options and data to guide treatment decisions may be limited. They have also been excluded from clinical trials of immunotherapies, such as nivolumab and pembrolizumab, with pharmacokinetic profiles more amenable to this population. Our study showed that anti-PD-1 agents may be a feasible alternative for this challenging population.

This study adds to a growing body of literature for patients with common, relevant medical co-morbidities that have been excluded from anti-PD-1 clinical trials. Recent data from a retrospective multicenter study evaluated the tolerability of ipilimumab in patients with baseline autoimmune diseases and similarly found that these therapies may be reasonable options for patients who are not traditionally included in standard clinical trials [12]. Other groups have reported their experiences with anti-PD-1 treatment in patients with prior organ transplant and ongoing immunosuppression, as well as patients with pre-existing hepatitis C and human immunodeficiency virus [13–15]. While these agents seemed largely safe and effective in patients with autoimmunity and hepatitis C, several cases of rejection have been observed in the organ transplant population. Interestingly, and perhaps surprisingly, ipilimumab appears to cause fewer rejection complications than does anti-PD-1 [16–18]. Further study in these clinically relevant populations is needed.

Our study has several other limitations. Patients received treatment at four different centers over multiple years, potentially resulting in variability in the quality of data capture. Patient numbers overall were limited, limiting the ability to draw definitive conclusions. Additionally, the patients in this cohort had a median ECOG PS of 1 with relatively few hospitalizations prior to treatment. As such, they may represent a relatively healthier group of patients with baseline organ dysfunction. Thus, this study may not fully capture safety and efficacy of anti-PD-1 in debilitated patients with more extensive co-morbidities. Because the patients in this study had various advanced malignancies, disease-specific conclusions cannot be made. However, this is the largest study of patients with solid tumors and significant baseline organ dysfunction treated with anti-PD-1 therapy to our knowledge.

## Conclusion

In conclusion, anti-PD-1 agents are associated with tolerable irAEs and relatively infrequent progressive organ dysfunction in patients with advanced malignancies and baseline organ dysfunction. Objective responses and prolonged PFS were observed. Prospective analyses and longer term follow-up are required to validate these findings.

## Abbreviations

ALT: Alanine aminotransferase
AST: Aspartate aminotransferase
CHF: Congestive heart failure
CTCAE: Common Terminology Criteria for Adverse Events
ECOG PS: Eastern cooperative oncology group performance status
eGFR: Estimated glomerular filtration rate
HIV: Human immunodeficiency virus
irAE: Immune related adverse event
NSCLC: Non-small cell lung cancer
OS: overall survival
PD-1: Programmed death receptor 1
PD-L1: Programmed death receptor ligand 1
PFS: Progression free survival
RCC: Renal cell carcinoma
RECIST: Response evaluation criteria in solid tumors
SCLC: Small cell lung cancer
